# Elevated Levels of CD4^+^CD25^+^FoxP3^+^ T Cells in Systemic Sclerosis Patients Contribute to the Secretion of IL-17 and Immunosuppression Dysfunction

**DOI:** 10.1371/journal.pone.0064531

**Published:** 2013-06-11

**Authors:** Xinjuan Liu, Na Gao, Mengtao Li, Dong Xu, Yong Hou, Qian Wang, Guohua Zhang, Qiuning Sun, Henghui Zhang, Xiaofeng Zeng

**Affiliations:** 1 Department of Rheumatology, Peking Union Medical College Hospital, Chinese Academy of Medical Science & Peking Union Medical College, Beijing, China; 2 Department of Dermatology, Peking Union Medical College Hospital, Chinese Academy of Medical Science & Peking Union Medical College, Beijing, China; 3 Peking University People's Hospital, Peking University Hepatology Institute, Beijing Key Laboratory of Hepatitis C and Immunotherapy for Liver Diseases, Beijing, China; University of Southern California, United States of America

## Abstract

**Objective:**

Immune imbalance between regulatory T (Treg) and Th17 cells is a characteristic of systemic sclerosis (SSc). The functional heterogeneity among Treg can be elucidated by separating Treg into different subsets based on the expression of FoxP3 and CD45RA. The aim of this study was to investigate the role of Treg subsets in the immune imbalance in naïve SSc.

**Methods:**

Peripheral blood mononuclear cells (PBMCs) of 31 SSc patients and 33 healthy controls were analyzed for the expression of CD4, CD25, CD45RA, CTLA-4, FoxP3, and IL-17 using flow cytometry. Treg immunesuppression capacity was measured in co-culture experiments. The expression of FoxP3, CTLA-4, IL-17A, and RORC mRNA was measured by real-time PCR.

**Results:**

The frequency of CD4^+^CD25^+^FoxP3^+^ Treg cells was significantly elevated in patients with SSc (3.62±1.14 vs 1.97±0.75, p<0.001) with diminished immunosuppression capacity. In SSc, the proportion of FoxP3^high^CD45RA^−^ activated Treg cells (aTreg) was decreased, the proportion of FoxP3^low^CD45RA^−^ T cells was increased, and the proportion of FoxP3^low^CD45RA^+^ resting Treg cells (rTreg) was decreased. The immune suppression capacity of aTreg and rTreg was diminished, while FoxP3^low^CD45RA^−^ T cells exhibited a lack of suppression capacity. The immune dysfunction of aTreg was accompanied by the abnormal expression of CTLA-4. Th17 cell numbers were elevated in SSc, FoxP3^low^CD45RA^−^ T cells produced IL-17, confirming their Th17 potential, which was consistent with the elevated levels of FoxP3^+^IL-17^+^ cells in SSc.

**Conclusion:**

A decrease in aTreg levels, along with functional deficiency, and an increase in the proportion of FoxP3^low^CD45RA^−^ T cells, was the reason for the increase in dysfunctional Treg in SSc patients, potentially causing the immune imbalance between Treg and Th17 cells.

## Introduction

Systemic sclerosis (SSc) is a complex autoimmune disease, for which effective treatments are not yet available. SSc is characterized by excessive collagen production resulting in skin and visceral fibrosis of various organs; however, the pathogenesis of SSc is not very clear. In general, the pathophysiology of SSc can be summarized as a combination of microvascular damage, slow-developing fibrosis, and an abnormal immune system. Immunological activity, especially of T lymphocytes, is considered to be a key stimulus in promoting the vascular abnormalities and fibrosis observed in SSc [1]. Many studies implicate the immune system in the pathology of SSc via the presence of autoantibodies and elevated cytokine levels. In addition, activated T lymphocytes, especially CD4^+^ T cells, are readily detected in the circulation and affected organs in SSc [2].

Regulatory T cells (Treg) are a subtype of CD4^+^ T cells that are indispensable for the maintenance of dominant self-tolerance and immune homeostasis. In general, Treg dysfunction is considered to be one of the major factors conferring risk of human autoimmune diseases [3]. However, recent studies failed to draw consistent conclusions regarding the role of Treg in autoimmune diseases, such as systemic lupus erythematosus (SLE) and rheumatoid arthritis (RA) [4]. Similarly, the relationship between Treg and SSc is another research focus. Most reports have shown that the ratio of Treg was elevated in the peripheral blood mononuclear cells (PBMCs) compartment in SSc, while some studies have reported normal or decreased Treg levels [5], [6], [7], [8], [9]. Nevertheless, it is generally thought that that immune suppression by Treg is abnormal in SSc due not only to a change in the frequency of Treg, but also to their dysfunction.

Th17 cells make up another CD4^+^ T cell subtype that secrete IL-17A and IL-17F, and induce inflammation [10]. Th17 cells play an important role in the development of autoimmune diseases, as elevated IL-17A levels are associated with SLE and RA. Similar to SLE and RA patients, Th17 and IL-17A levels are higher in SSc patients compared to healthy individuals [11], [12]. Interestingly, it seems that both Treg and Th17 levels are elevated in SSc. The opposing role of Th17 and Treg cells is evident not only in their immune modulatory functions, but also in their differentiation [13]. In fact, immune imbalance between Th17 and Treg cells is a well-documented characteristic of SSc [14], [15].

The transcription factor forkhead box P3 (FoxP3) is an important marker and functional molecule for Treg. Recent studies have shown that human CD4^+^FoxP3^+^ T cells are not homogeneous in their gene expression. Sakaguchi et al. defined the subtypes of Treg based on the expression of FoxP3 and CD45RA, including subtypes such as CD4^+^CD25^+^FoxP3^low^CD45RA^+^ (FrI), CD4^+^CD25^high^FoxP3^high^CD45RA^−^ (FrII), and CD4^+^CD25^+^FoxP3^low^CD45RA^−^ (FrIII). The FrII subtype consists of activated Treg (aTreg), which have suppressive function. The FrI subtype consists of resting Treg (rTreg), which can convert to aTreg, while the FrIII subtype consists of T cells that are not suppressors, can produce IL-17, and hence have Th17 potential [16].

In this study, we examined the subsets of Treg in SSc. We found that the percentage of FrI, FrII, and FrIII subsets were abnormal in SSc, which associated with CLTA-4 (cytotoxic T-lymphocyte antigen-4), an important negative functional molecular in Treg. And there were a subset of CD4^+^ T cell, which were both positive of FoxP3 and IL-17, maybe one subset in the intermediate state between Treg and Th17, resulted in CD4^+^CD25^+^FoxP3^+^ cells increased in patients of Treg cells.

## Materials and Methods

### Study Subjects

Thirty-one Chinese patients with SSc from Peking, China fulfilled the American College of Rheumatology (ACR) or LeRoy and Medsger criteria [17], [18]. Patients in this study had new-onset SSc without any previous treatment (Table 1). Thirty-three age- and sex-matched healthy volunteers were enrolled as controls. This study was approved by the Medical Ethics Committee of Peking Union Medical College Hospital and the Ethics Committee of EUSTAR. All participants have provided their written informed consent to participate in this study.

**Table 1:** Participant demographics and clinical characteristics.

Variable	SSc patients	Healthy controls
Number	31	33
Sex (male:female)	4∶27	5∶28
Age (years)*	41.48±12.28	40.36±10.13
Disease duration (years)*	3.32±3.1	
dcSSc/lcSSc	16/15	
ANA positivity (%)	28 (90.32)	
Anti-Scl-70 positivity (%)	13 (41.94)	
ACA positivity (%)	5 (16.13)	
PAH (%)	3 (9.68)	
mRSS at inclusion*	16.68±3.12	
6MWD (m)*	449.17±86.59	

dcSSc: diffuse cutaneous SSc; lcSSc: limited cutaneous SSc; ANA: antinuclear antibody; Anti-Scl70: antitopoisomerase I antibody; ACA: anticentromere antibody; PAH: pulmonary arterial hypertension; mRSS: modified Rodnan skin score; 6MWD: 6-minute walk distance. * indicates values reported as mean ± standard deviation.

### Antibodies

Anti-human mouse antibodies (conjugated with FITC, PE, PerCP, allophycocyanin (APC), Alexa Fluor 488, and PE-cyanin 7 (PE-Cy7), as indicated) used in this study were: CD4-PerCP (OKT4, Biolegend), CD4-PE-Cy7 (RPA-T4, BD Pharmingen), CD25-APC (BC96, eBioscience), FoxP3-PE, FoxP3-PE-Cy7, FoxP3-Alexa Fluor 488 (PCH101, respectively, eBioscience), CD45RA-FITC, CD45RA-PE, CTLA-4-PE, CD69-PE, CD279-PE (L48, HI100, BNI3, FN50,MIH4,respectively; BD Pharmingen), GITR-PE (eBioAITR, eBioscience), IL-17-APC (eBio64DEC17, eBioscience), and IL-17-Alexa Fluor 488 (N49-653, BD Pharmingen). In all experiments, a control antibody of the respective IgG isotype was included.

### Cell phenotypic analysis by flow cytometry

Peripheral blood of patients and healthy individuals was collected and peripheral blood mononuclear cells (PBMCs) were prepared by Ficoll gradient centrifugation. PBMCs were washed in PBS containing 2% fetal calf serum and incubated with CD4, CD25, CD45RA, CTLA-4, CD69, CD279, and GITR antibodies in the dark at 4°C for 30 min. Subsequently, intracellular FoxP3 staining was performed according to the manufacturer's instructions. For detection of intracellular cytokine production, after stimulated with 20 ng/mL PMA and 1000 ng/mL ionomycin in the presence of Golgi-Stop (BD Biosciences) for 5 hr, cells were incubation with CD4, CD25, or CD45RA and then stained with antibodies against FoxP3 and IL-17 after fixation and permeabilization (eBioscience). Stained cell images were acquired using a FACSCalibur flow cytometer (Becton Dickinson) and analyzed with FlowJo v.7.6.4 software (Tree Star).

### Cells sorting and Suppression Assay

PBMCs were stained with CD4, CD25, and CD45RA antibodies, and sorted with a FACS AriaII flow cytometer (Becton Dickinson). RPMI 1640 medium supplemented with 10% fetal bovine serum, 100 IU/mL penicillin, and 100 mg/mL streptomycin was used for cell culture. Ten-thousand responder CD4^+^CD25^−^ cells from a healthy donor were labeled with 1 μM CFSE (Invitrogen) and co-cultured with 10^4^ unlabeled Treg cells or subsets of Treg cells from patients and control donors at a suppressor:responder cell ratio of 1∶1. Hundred thousand PBMCs from the same healthy donor were incubated with 25 μg/mL mitomycinC (MMC) at 37°C for 30 min, and added to the co-cultures as feeder cells. Co-cultures were stimulated with 0.5 μg/mL anti-CD3 (OKT3, BD Pharmingen) and 1 μg/mL anti-CD28 (CD28.2, BD Pharmingen) antibodies in 96-well round-bottom plates supplemented with RPMI medium. Proliferation of CFSE-labeled cells was assessed by ﬂow cytometry after 96 hr.

### Real-time PCR

Total RNA was extracted from 1×10^4^ sorted Treg subsets from patients and healthy controls (RNeasy Micro Kit, QIAGEN), then reverse transcribed to obtain cDNA (PrimeScript RT reagent Kit, TaKaRa). Real-time PCR was performed with a SYBR green assay (SYBR Premix Ex TaqTM, TaKaRa) using the ABI 7900HT system (Applied Biosystems). Primer used were: FoxP3-F, 5'-CCACTCACCTCACTCCCATT-3'; FoxP3-R, 5'-GCAGTTTATTGGGGACGGTA-3'; CTLA-4-F, 5'-GCTGGTGGTATCTGAGTTGAC-3'; CTLA-4-R, 5'-ACCTGCTGCCTTCTTCTGT-3'; IL-17A-F, 5'-CACTGCTACTGCTGCTGAG-3'; IL-17A-R, 5'-GGTGAGGTGGATCGGTTGT-3'; RORC-F, 5'-GAGGAAGTCCATGTGGGAGA-3'; RORC-R, 5'-CACTTCCATTGCTCCTGCTT-3'; GAPDH-F, 5'-TCGGAGTCAACGGATTTGGTC-3'; and GAPDH-R, 5'-GCCATGGGTGGAATCATATTGG-3'.

### Statistical Analysis

Data were analyzed with the SPSS v.17.0 statistics package (IBM, USA). Variables were summarized as counts and percentages or as medians and ranges. The independent samples t-test and the nonparametric Mann-Whitney U test were used to compare data between the groups. P values less than 0.05 were considered to be statistically significant.

## Results

### CD4^+^CD25^+^FoxP3^+^ Tregs are elevated, yet dysfunctional in SSc patients

**Figure 1:**
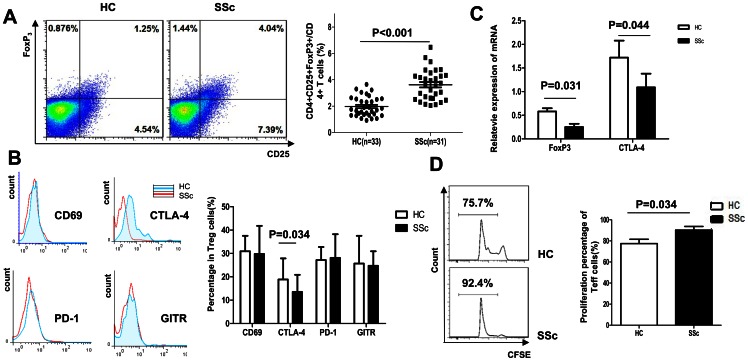
CD4^+^CD25^+^FoxP3^+^ regulatory T cells in patients with SSc. (A) A representative FACS analysis of CD25^+^ FoxP3^+^ cells gated by CD4^+^ T cell subsets in healthy controls and SSc patients, comparing the percentage of CD4^+^CD25^+^FoxP3^+^ cells among the CD4^+^ T cells (B) Percentage of CD69-, CTLA-4-, PD-1-, and GITR-expressing CD4^+^CD25^+^FoxP3^+^ cells in healthy controls and patients analyzed by flow cytometry (C) The expression of FoxP3 and CTLA-4 mRNA in healthy individuals and SSc patients abtained with real-time PCR (D) Flow cytometry analysis of CFSE-labeled responder T cells and dividing cells in SSc patients and healthy individuals. The CFSE labeled responder cells under the bin indicate daughter cell populations which have subsequently lost half of their CFSE signal with each division round.The percentage of proliferative responder T cells co-cultured with Treg cells (1∶1) from control (n = 5) and SSc patients (n = 5) is shown.

FoxP3 is considered the best and most specific marker for Treg. The frequency of CD4^+^CD25^+^FoxP3^+^ Treg among PBMCs in 31 SSc patients and 33 healthy controls was assessed by flow cytometry. The frequency of CD4^+^CD25^+^FoxP3^+^ Treg cells was significantly elevated in patients (3.62±1.14) compared to healthy controls (1.97±0.75, p<0.001; Figure 1A).

We also compared the phenotypic Treg markers, CLTA-4, PD-1, GITR, and CD69, between SSc patients and healthy individuals. CLTA-4 is a protein receptor predominantly found on T lymphocytes and plays an important role in Treg function [19]. Programmed death-1 (PD-1) is a surface receptor critical for the regulation of T cell function during immunity and tolerance, playing a role in long-term tolerance maintenance [20]. Glucocorticoid-induced tumor necrosis factor receptor related protein (GITR) has been used as a marker of suppressor T cells and is involved in suppressor activity, though its function is controversial [21]. CD69 potentially reflects Treg cell activation, and the CD69-dependent induction of TGFβ can influence the development of a subset of adaptive Treg cells [22]. In this study, the level of CTLA-4 was lower in the CD4^+^CD25^+^FoxP3^+^ cells of SSc patients compared with the cells of healthy controls (p = 0.034), but not the levels of CD69 (p = 0.104), PD-1 (p = 0.268), and GITR (p = 0.296; Figure 1B). Interestingly, real-time PCR results indicted that FoxP3 and CTLA-4 mRNA levels were lower in Treg cells of SSc patients compared to the levels in Treg cells of healthy individuals (p<0.05, p<0.05, respectively; Figure 1C).

Although the frequency of Treg was higher in SSc patients, the immunosuppression capacity of Treg was diminished (p = 0.034), based on the proliferative status of the responder T cells of one healthy donor co-cultured with the Treg from SSc patients or other healthy donors. The proliferation of Treg was not different between SSc patients and control individuals (p = 0.142; data not shown). Together, these results indicate that even though the CD4^+^CD25^+^FoxP3^+^ Treg levels were elevated in SSc patients, the suppression function of Treg was deficient (Figure 1D).

### SSc patients have fewer CD4^+^CD25^high^FoxP3^high^CD45RA^−^ aTreg cells (FrI) and more CD4^+^CD25^+^FoxP3^low^CD45RA^−^ cells (FrIII)

**Figure 2:**
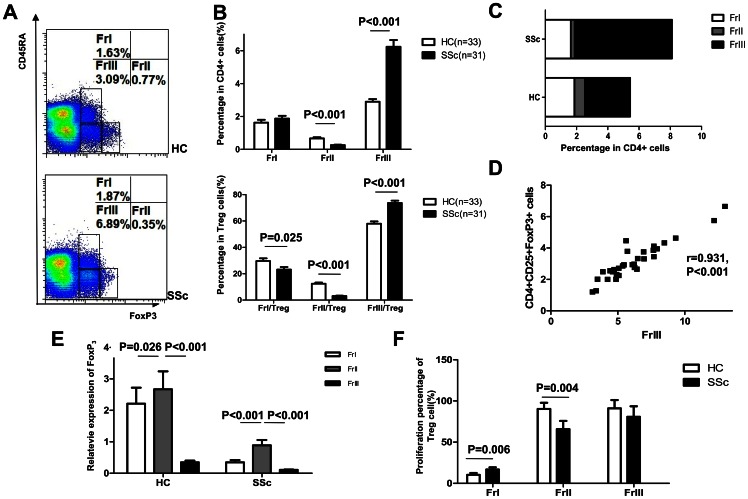
The analysis of Treg subpopulations of SSc patients with regard to the expression of CD45RA. (A) A representative flow cytometry analysis of Treg subpopulations gated by CD4^+^ T cell subsets (B) Percentages of FrI, FrII, and FrIII cells among CD4^+^ T cells or CD4^+^FoxP3^+^ T cells in healthy controls and SSc patients (C) Proportion of FrI, FrII, and FrIII subsets in the Treg compartment in healthy individuals and SSc patients (D) The correlation between increasing FrIII cells and CD4^+^CD25^+^FoxP3^+^ cells in SSc patients (E) FoxP3 mRNA levels in FrI, FrII, and FrIII cells in healthy controls and patients obtained with real-time PCR (F) Flow cytometry analysis of CFSE-labeled Treg subsets cells in SSc patients and healthy individuals.The proliferation of FrI, FrII, and FrIII cells in patients (n = 5) compared to that in controls (n = 5).

Next, we wanted to assess the proportion of FrI, FrII, and FrIII Treg subsets in SSc patients. We found that the proportion of FrII cells among all CD4^+^ cells was decreased in SSc patients compared to healthy controls (0.25±0.16 vs 0.66±0.41, respectively; p<0.001). Similarly, the proportion of FrII cells among the CD4^+^CD25^+^FoxP3^+^ Treg cells (FrII/Treg) was also decreased in SSc patients compared to healthy individuals (12.42±5.23 vs 30.01±1.74, respectively; p<0.001). On the other hand, the proportions of FrIII cells were markedly increased (6.23±2.29 vs 2.90±0.91 among CD4^+^, p<0.001; 73.71±9.62 vs 57.96±9.90 among Treg, p<0.001) in SSc patients compared to healthy controls, respectively. The proportion of FrI cells among Treg cells (FrI/Treg) was decreased in SSc patients compared to healthy individuals, but there was no significant difference in FrI cell levels in the CD4^+^ compartments of patients and controls (23.19±10.60 vs 29.63±11.77 among Treg, p = 0.025; 1.87±0.94 vs 1.63±0.97 among CD4^+^, p = 0.320)(Figure 2A–B). Furthermore, FrIII was the major subset of Treg in both SSc patients and healthy controls. In the patients, the increased FrIII subset was positively correlated to the increase in CD4^+^CD25^+^FoxP3^+^ Treg cells (r = 0.931, p<0.001; Figure 2C–D).

The expression level of FoxP3 mRNA in the three Treg subsets was determined by real-time PCR. FoxP3 mRNA levels were higher in FrII than in FrI and FrIII (p<0.05) in patients and healthy controls alike. Interestingly, FoxP3 mRNA levels in FrI, FrII, and FrIII subsets were lower for SSc patients than for healthy individuals (p<0.05; Figure 2E).

Furthermore, the proliferation of the FrII cells of patients was lower than those of healthy controls (p = 0.004). Although the frequency of rTreg in SSc patients was similar to that of healthy individuals, the proliferation of patient rTreg was increased compared to the proliferation of rTreg of controls (p = 0.006). There was no difference in the proliferation of FrIII cells from patients and controls (p = 0.154; Figure 2F).

### SSc patients have CD4^+^CD25^+^FoxP3^low^CD45RA^−^ cells lacking suppression function and dysfunctional aTregs

**Figure 3:**
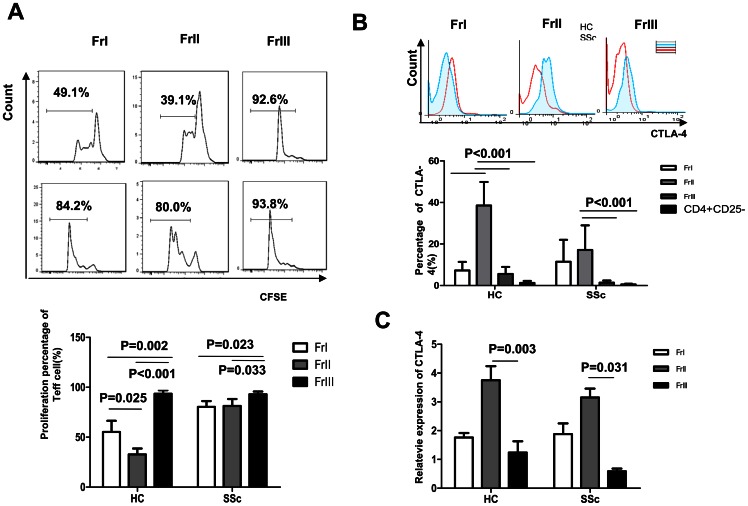
The immunosuppression capacity of Treg subsets cells in SSc patients and healthy individuals. (A) A representative flow cytometry analysis of the immunosuppression capacity of Treg subsets. CFSE-labeled effector T cells and dividing cells were analyzed by flow cytometry. The percentage of proliferating responder T cells co-cultured with different subsets of Treg cells (1∶1) from SSc patients (n = 5) and healthy controls (n = 5) is shown (B) Analysis of CTLA-4 presentation in each Treg subset in SSc patients and control individuals by flow cytometry (C) The expression of CTLA-4 mRNA in Treg cells from healthy controls and patients.

To assess the in vitro suppressive potency of each fraction, we sorted the three subsets of Treg from the PBMCs of SSc patients of and health controls and co-cultured the Treg subsets with the same responder T cells. The FrI and FrII subsets isolated from healthy individuals suppressed the proliferation of responder T cells, but the FrIII subset had no suppression capacity (p = 0.002, p<0.001, respectively). The suppression capacity of FrI was weaker than that of FrII (p = 0.025). On the other hand, the suppression capacity of the FrIII Treg subset isolated from SSc patients was reduced compared to the capacity of the other two subsets (p = 0.023, p = 0.033, respectively), but there was no difference in the suppression capacities of SSc FrI and FrII subsets (p = 0.974). Furthermore, the suppression capacity of FrI (p = 0.011) and FrII (p<0.001) subsets isolated from SSc patients was diminished compared to that of the FrI and FrII subsets of healthy individuals; however, the FrIII subsets from SSc patients and healthy controls didn't significantly differ in suppression capacity (p = 0.873; Figure 3A).

CLTA-4 plays an important role in Treg function, which functions at the cell surface, yet is primarily localized in intracellular vesicles of resting T cells [23]. Cell surface expression of CTLA-4 rapidly increases during T cell activation. Unlike other T cells, Treg constitutively express CTLA-4 at the cell surface [24]. We found that CTLA-4 surface expression was decreased in Treg of SSc patients. We also found that CTLA-4 expression was lower in the FrII (p<0.001) and FrIII (p = 0.001) subsets from SSc patients compared to that of FrII and FrIII subsets from healthy individuals. However, CTLA-4 expression did not differ between the FrI subsets (p = 0.253) of patients and controls. CTLA-4 surface expression in the FrII subset was markedly higher than in the FrI and FrIII subsets and also higher than in the CD4^+^CD25^−^ compartment of healthy donors (p<0.001). Although CTLA-4 surface expression in the FrII subset was higher than in the FrI and CD4^+^CD25^−^ compartment (p<0.001), there was no difference in CTLA-4 surface expression between FrI and FrII cells from SSc patients (p = 0.412; Figure 3B).

In order to understand the possible cause of the differences we observed in the cell surface expression of CTLA-4, we investigated CTLA-4 gene expression levels in each Treg subset. CTLA-4 mRNA levels in the FrII and FrIII subsets were lower in SSc patients than in health controls (p<0.05); however, there was no difference in CTLA-4 mRNA levels between the SSc and healthy FrI subsets (Figure 3C). Interestingly, CTLA-4 mRNA levels were higher in the FrII subsets of patients and healthy individuals than in the FrIII subsets of corresponding groups (p<0.05), but CTLA-4 gene expression was similar between the FrI and FrII subsets in both groups.

### CD4^+^CD25^+^FoxP3^low^CD45RA^−^ non-Treg cells secrete IL-17 in SSc patients

**Figure 4:**
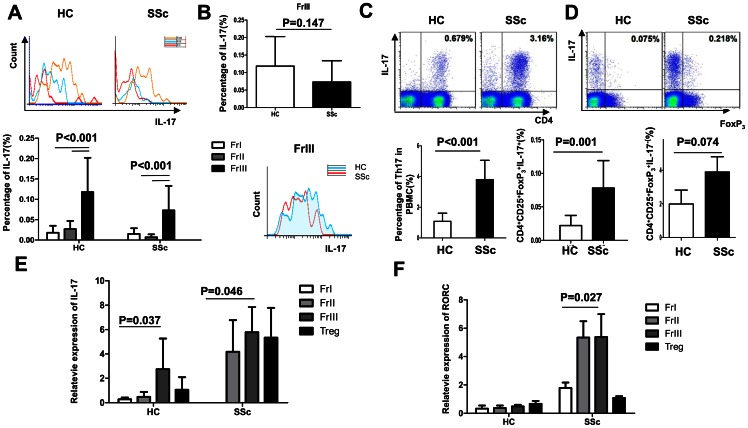
Expression of IL-17 in Treg cells in SSc. (A) IL-17 presentation in cells from the Treg subsets isolated from patients and control individuals by flow cytometry (B) The expression of IL-17 in the FrIII subset in SSc and control individuals by flow cytometry (C) The frequency of Th17 cells among Tregs (D) Flow cytometry analysis of the FoxP3^+^IL-17^+^ cells gated among the CD4^+^CD25^+^ cells in patients and healthy controls, comparing the frequency of CD4^+^CD25^+^FoxP3^+^IL-17^+^ and CD4^+^CD25^+^FoxP3^+^IL-17^−^ cells (E) Comparison of IL-17A mRNA expression of in Treg subsets isolated from SSc patients and control individuals (F) Comparison of RORC mRNA expression in Treg subsets isolated from patients and healthy controls.

We found that CD4^+^CD25^+^FoxP3^low^CD45RA^−^ non-Treg cells, the major part of the CD4^+^CD25^+^FoxP3^+^ Treg compartment, were increased in SSc patients. Considering that these non-Treg cells have no suppression capacity, we can surmise that the FrIII cells are non-suppressive T cells that produce IL-17, and hence have Th17 cell potential. Indeed, we found that IL-17 levels of the FrIII subset were markedly higher than that of the FrI and FrII subsets both in health donors and in SSc patients. However, there was no difference in IL-17 levels between the patient and control FrIII subsets (0.073±0.006 vs. 0.118±0.084, respectively; p = 0.147; Figures 4A and 4B). Along the same lines, CD4^+^IL-17^+^ cells, which represent Th17 cells, were higher in number in SSc patients than in healthy controls (3.80±1.26 vs. 1.08±0.53, respectively; p<0.001). CD25^+^FoxP3^+^IL17^+^ cells among CD4^+^ cells were also more numerous in SSc patients than in healthy individuals (0.069±0.038 vs. 0.022±0.015, respectively; p = 0.001), but SSc patients and controls had similar levels of CD25^+^FoxP3^+^IL17^−^ cells (3.16±0.68 vs. 1.99±0.83, respectively; p = 0.074; Figures 4C and 4D).

RORC is an important marker and functional molecule for Th17 cells. We also measured the IL-17A and RORC mRNA expression levels in each Treg subset. Our results showed that IL-17A expression levels were higher in the FrIII subset than in the FrII subset in both SSc patients and healthy controls (p<0.05). There was scarcely any IL-17A expression in the FrI fraction of either SSc patients or healthy controls. IL-17A levels were higher in the FrII, FrIII, and Treg compartments of SSc patients than in those of control individuals. RORC expression levels were similar between the SSc FrII and FrIII subsets and higher than in the SSc FrI subset (p = 0.027). On the other hand, RORC was rarely expressed in the Treg of healthy controls, and hence RORC levels were higher in SSc patients than in healthy individuals for each Treg subset (p<0.05; Figures 4E and 4F).

## Discussion

Treg cells are suppressor T cells, which downregulate the immune system and are associated with autoimmune diseases. However, so far, a decrease in Treg cells in autoimmune diseases, such as SLE, RA and SSc, has not been reported consistently. In fact, SSc patients have higher amounts of both Treg and Th17 cells, and an immune imbalance between Th17 and Treg cells is a characteristic of SSc. In this study, we first verified the previous research results in our group, showing that CD4^+^CD25^+^FoxP3^+^ Treg levels are elevated in patients with naïve SSc; however, interestingly, these CD4^+^CD25^+^FoxP3^+^ Tregs lack their normal immune suppression capacity. Recent studies have shown that human CD4^+^FoxP3^+^ T cells are not homogeneous in gene expression, phenotype, and immune suppression function. This functional heterogeneity can be elucidated by separating Treg cells into three subsets based on the expression of FoxP3 and CD45RA [16], [25]. Based on these previous findings, we systemically evaluated Treg cells and their subsets in SSc patients. Our results confirmed that the increase in Treg cells was mainly due to elevated CD4^+^CD25^+^FoxP3^low^CD45RA^−^ (FrIII) cell number. These FrIII cells were not functionally suppressive. Furthermore, the aTreg cells (FrII), which contribute most to the immune suppression by Treg, were decreased in SSc patients.

Most rTreg cells (FrI) are in a resting state, and once stimulated, they differentiate into aTreg and proliferate. The aTreg cells develop from activated and proliferating rTreg cells and die after fulfilling their immune suppression function. Furthermore, aTreg cells control rTreg cell expansion via a feedback loop, contributing to the maintenance of immune balance. We not only found that aTreg levels in SSc patients were lower than in healthy individuals, but also that these cells proliferated less in SSc patients, which suggests that more activated and proliferating rTregs would be needed in these patients. Indeed, our results show that rTreg proliferation was increased in SSc patients, while it was minimal in healthy individuals.

The role of Treg cells in autoimmune disease is thought to be their inability to maintain immune tolerance, resulting in proliferation of effector T cells. We found that the CD4^+^CD25^+^FoxP3^low^CD45RA^−^ cells isolated from SSc patients, though elevated in number, had no suppressive function *in vitro*. Instead, FoxP3^high^ Treg cells were the main functional suppressive Tregs isolated from SSc patients, which were not only fewer in number compared to the Tregs isolated from healthy controls, but also dysfunctional, as judged by their CTLA-4 deficiency. As a negative regulator of T cells, CTLA-4 has an important role in the function of Treg through multiple mechanisms. The role of CTLA-4 in the suppressive function of Tregs is to compete with CD28 on effector cells, which is mediated by CTLA-4-dependent down-regulation of CD80 and CD86 on APCs. FoxP3^+^CTLA-4^+^ cells are suppressive, but FoxP3^+^CTLA-4^−^ cells are not [19]. This represents the cell-extrinsic mechanism of CTLA-4 on Tregs [26]. Moreover, CTLA-4 is required for TGF-β to induce FoxP3 and generate suppressor T cells [27], [28]. In general, Tregs inhibit the function and proliferation of effector cells by cell-cell contact via the expression of surface TGF-β. CTLA-4 signaling in CD25^+^ T cells enhances the suppressive signal by concentrating TGF-β at the point of cell-cell contact [29], [30]. In healthy individuals, the FrII subset highly expresses CTLA-4, but FrI and FrIII hardly express it. We found that the expression of CTLA-4 was decreased in both Treg and the FrII subset in SSc patients. It has been reported that polymorphisms and the expression of CTLA-4 are associated with SSc susceptibility [31], [32], [33], [34], [35]. Moreover, TGF-β levels in Treg cells are lower in SSc patients [5]. In addition, FrI hardly express CTLA-4 in both healthy and SSc patients. In general, CTLA-4 is expressed on the surface of activated cells, but resides in endocytic vesicles and secretory granules of resting cells [36]. Thus, the mechanism of FrI dysfunction will require further research.

Since we confirmed that the elevation in Treg cells was mainly due to an elevation in CD4^+^CD25^+^FoxP3^low^CD45RA^−^ cell number, and that this subset lacked suppression capacity, we further investigated the characteristics and functionality of cells of this subtype. Since FrIII cells express the highest level of IL-17 among the Treg subsets, we conjectured that FrIII cells co-express FoxP3 and IL17. Meanwhile, both previous studies [25], [37] and our group found that FrIII lack suppressive function on the proliferation of effector T cells. Therefore, FrIII cells can be thought of as FoxP3^+^ non-Treg cells. Moreover, we also confirmed that Th17 cells were elevated in these patients, consistent with the majority of previous studies [12], [38], [39], [40], [41], [42]; however, one recent report demonstrated contrary results [43]. Upon removing FoxP3^+^IL-17^+^ cells from the CD4^+^CD25^+^FoxP3^+^cell population, we further confirmed that there was no difference in the CD4^+^CD25^+^FoxP3^+^IL-17^−^ cell population between SSc patients and healthy individuals, which also confirmed our hypothesis. As a result, we concluded that despite augmentation of the FoxP3^+^ cells in SSc patients, the majority consist of non-suppressive FrIII cells.

It is important to understand how FrIII might be involved in SSc pathogenicity. Recently, researchers have found that different subsets of effector T cells characterized by special cytokines and unique transcription factors are not stable lineages differentiated from naïve T cells. Studies about plasticity of CD4^+^FoxP3^+^ cells found that, upon stimulation by inflammatory cytokines, CD4^+^FoxP3^+^ cells downregulate FoxP3 expression and produce cytokines such as IL-17 and IFN-γ, and that these “ex-Treg” are present in multiple inflammatory settings [37], [44], [45], [46]. Interestingly, we found that FrIII cells possess some characteristics of these ex-Tregs, so we propose that the FrIII compartment may represent a stage in the development of CD4^+^FoxP3^+^ plasticity.

Consistent with our results, other groups have identified T cells that co-express FoxP3 and IL-17, which represent a transitional phase in the conversion process from Treg to Th17 cells [47], [48]. For this subset of cells, the RORγt-driven inflammatory profile might play a dominant role. Our results indicate that the FrIII subset is the provider of FoxP3^+^IL17^+^ cells, which can secrete IL-17. We propose that the FrIII subset is involved in the inflammatory process in SSc; however, further work is needed to confirm this hypothesis.

Importantly, both FoxP3^+^ cells and IL17^+^ cells are elevated in number in SSc patients, which suggests that an immune imbalance between Th17 and Treg cells is a characteristic of SSc. Here we provide novel insights into the subsets of Treg cells in SSc. A decrease in the number and functionality of aTreg and an increase in FoxP3^low^CD45RA^−^ T cells was the reason for the elevation in the number and dysfunction of Tregs in SSc, which may be the reason for the immune imbalance observed in SSc. Increased CD4^+^CD25^+^FoxP3^+^ cells were not the effective regulatory T cells. Instead, the main Treg compartment was composed of non-suppressive CD4^+^CD25^+^FoxP3^low^CD45RA^−^ cells, which accounted for the FoxP3^+^IL17^+^ cells. Further concerns about Treg cells in patients with SSc should discern aTreg from CD4^+^CD25^+^FoxP3^low^CD45RA^−^ non-Treg cells.
